# EnrichNet: network-based gene set enrichment analysis

**DOI:** 10.1093/bioinformatics/bts389

**Published:** 2012-09-03

**Authors:** Enrico Glaab, Anaïs Baudot, Natalio Krasnogor, Reinhard Schneider, Alfonso Valencia

**Affiliations:** ^1^Luxembourg Centre for Systems Biomedicine (LCSB), University of Luxembourg, L-4362 Esch-sur-Alzette, Luxembourg; ^2^Luminy Institute of Mathematics (IML), Université d'Aix-Marseille, 13288 Marseilles, France; ^3^Interdisciplinary Computing and Complex Systems (ICOS) Research Group, University of Nottingham, Nottingham NG8 1BB, UK; ^4^Structural Biology and Biocomputing Program, Spanish National Cancer Research Centre (CNIO), E-28029 Madrid, Spain

## Abstract

**Motivation:** Assessing functional associations between an experimentally derived gene or protein set of interest and a database of known gene/protein sets is a common task in the analysis of large-scale functional genomics data. For this purpose, a frequently used approach is to apply an over-representation-based enrichment analysis. However, this approach has four drawbacks: (i) it can only score functional associations of overlapping gene/proteins sets; (ii) it disregards genes with missing annotations; (iii) it does not take into account the network structure of physical interactions between the gene/protein sets of interest and (iv) tissue-specific gene/protein set associations cannot be recognized.

**Results:** To address these limitations, we introduce an integrative analysis approach and web-application called EnrichNet. It combines a novel graph-based statistic with an interactive sub-network visualization to accomplish two complementary goals: improving the prioritization of putative functional gene/protein set associations by exploiting information from molecular interaction networks and tissue-specific gene expression data and enabling a direct biological interpretation of the results. By using the approach to analyse sets of genes with known involvement in human diseases, new pathway associations are identified, reflecting a dense sub-network of interactions between their corresponding proteins.

**Availability:** EnrichNet is freely available at http://www.enrichnet.org.

**Contact:** Natalio.Krasnogor@nottingham.ac.uk, reinhard.schneider@uni.lu or avalencia@cnio.es

**Supplementary Information:** Supplementary data are available at *Bioinformatics* Online.

## 1 MOTIVATION

The analysis of functional genomics data from high-throughput experiments often involves the assessment of potential functional associations between a gene or protein set of interest, e.g. differentially expressed genes in a microarray study and known gene/protein sets representing cellular processes and pathways. To identify and prioritize these putative associations, a wide range of enrichment analysis tools have been developed in recent years, including three basic types of methods (see Huang *et al.* (2009) for a more comprehensive review):

1. Over-representation analysis (ORA) techniques, assessing the statistical overrepresentation of a user-defined, pre-selected gene/protein list of interest in a reference list of known gene/protein sets using a statistical test, e.g. the one-sided Fisher's exact test or the hypergeometric distribution.
2. Gene set enrichment analysis (GSEA) methods, which in contrast to classical annotation enrichment analyses incorporate expression level measurements from an unfiltered dataset, including non-parametric approaches such as GSEA (Subramanian *et al.*, 2005), Catmap (Breslin *et al.*, 2004), ErmineJ (Lee *et al.*, 2005) and GeneTrail (Backes *et al.*, 2007) and parametric approaches such as PAGE (Kim and Volsky, 2005), MEGO (Tu *et al.*, 2005), FatiScan (Al-Shahrour *et al.*, 2007) and GAGE (Luo *et al.*, 2009).
3. Integrative and modular enrichment analysis (MEA) approaches (Huang *et al.*, 2009), which account for dependencies between genes and proteins inferred from biological networks and ontology graphs (e.g. Ontologizer (Bauer *et al.*, 2008) and GeneCodis (Carmona-Saez *et al.*, 2007)) or by combining multiple types of annotations (e.g. DAVID (Dennis Jr *et al.*, 2003)).

Most of these approaches provide a ranking list of known gene/protein sets as output, scoring the evidence for their association with a user-defined target gene/protein list of interest. Although these prioritized, putative functional associations are a useful starting point for further experimental validation and analysis, they also have the following major limitations (among others):

- ORA techniques tend to have low discriminative power (for a target gene set, several reference gene sets receive the same or similar significance scores, e.g. see Table 1) and the scores vary considerably with small changes in the overlap size.
- Functional information captured in the graph structure of a molecular interaction network connecting the gene/protein sets of interest is disregarded.
- Genes and proteins in the network neighbourhood, in particular those with missing annotations, are not taken into account.
- The recognition of tissue-specific gene/protein set associations is often statistically infeasible.

**Table 1.** Xd-score ranking table for the top 20 functional associations between genes mutated in gastric cancer and pathways in the BioCarta database (see also the correlation plot for the same dataset in Fig. 1)

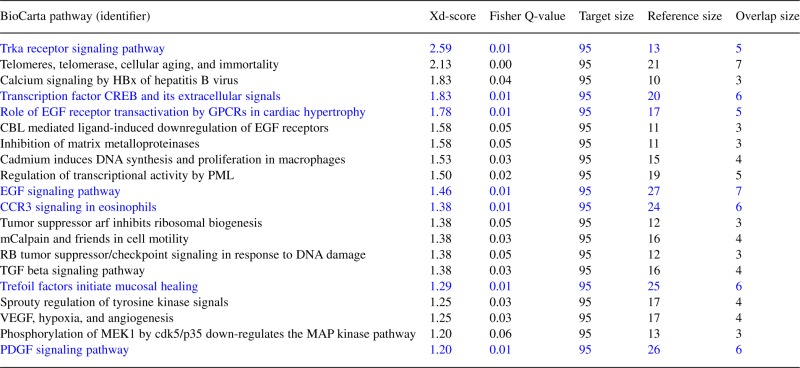

Pathways with the same Fisher Q-value 0.01 but different Xd-scores are highlighted in blue colour.

These limitations are mutually enhancing, since the combination of low robustness in the scoring of gene/protein set associations and low interpretability of the results increases the difficulty of deriving new biological insights from the analysis. We therefore propose to tackle all these problems simultaneously by introducing EnrichNet, a new integrative enrichment analysis method.

EnrichNet combines a novel graph-based statistic, developed to exploit information from the molecular network structure connecting two gene/protein sets, with a new interactive visualization of network sub-structures. This combined network analysis and visualization enables a direct molecular interpretation of how a user-defined set of genes/proteins is related to a gene/protein set of known function. Based on a previous work on combining network and pathway analysis methods (Glaab *et al.*, 2010*a*, *b*) the integrated data sources (molecular interaction data, cellular pathway data and tissue-specific gene expression data) and analysis techniques (graph-based statistical analysis and force-directed layout generation for sub-networks) have been designed to build on each other to provide a clearer and more detailed understanding of gene/protein set functional associations. To further facilitate the analysis, a complete implementation of the integrative approach is made freely available as a public web application with an exposed programmatic API (www.enrichnet.org).

In the following, we explain the EnrichNet methodology in detail and show example results obtained from its application on gene sets known to be associated with complex diseases.

## 2 SYSTEM AND METHODS

### 2.1 General workflow

A gene/protein list analysis with EnrichNet can be performed in a fully automated fashion and does not require any parameter settings.

#### 2.1.1 Input

The only required input is a list of 10 or more human gene or protein identifiers and the selection of a database of interest (KEGG (Kanehisa *et al.*, 2006), BioCarta (Nishimura, 2001), WikiPathways (Pico *et al.*, 2008), Reactome (Joshi-Tope *et al.*, 2005), PID (Schaefer *et al.*, 2009), InterPro (Apweiler *et al.*, 2001) or GO (Ashburner et al., 2002), from which reference gene/protein sets will be extracted.

#### 2.1.2 Processing

After mapping the target and reference datasets onto a genome-scale molecular interaction network (two default networks are available, alternatively a user-defined network can be provided, but the availability of sufficient interaction data for the mapping of the target and reference datasets has to be ensured, see implementation details in the Supplementary Materials) a network analysis procedure is applied, consisting of two basic steps: a procedure to score the distances between the mapped target gene set and reference datasets in the network using a random walk with restart (RWR) algorithm and the comparison of these scores against a background model. This random walk and scoring procedure is explained in detail in the following section.

#### 2.1.3 Output

As a final output, a ranking table of the reference datasets (e.g. cellular pathways, processes and complexes) is generated, including their network-based association scores and tissue-specific association scores across 60 human tissues. For each pathway, a hyperlink enables the user to generate an interactive graph-based visualization of the sub-network representing the analysed datasets in the molecular interaction network. The user can explore this network by zooming into it, searching and highlighting specific genes/proteins and retrieving additional annotations and topological information by clicking on a node of interest (see tutorial on the web page for details).

### 2.2 Algorithm

**Fig. 1.**
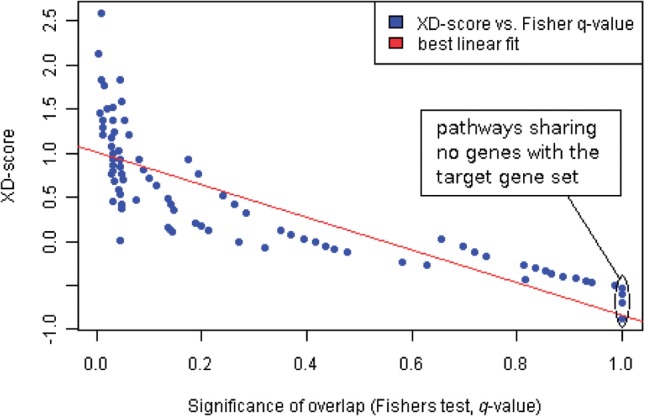
Regression plot: Xd-scores versus significance-of-overlap scores (Fisher's test, *q*-values), computed for the comparison of gastric cancer mutated genes against gene sets from the BioCarta database (absolute Pearson correlation: 0.93). Non-overlapping dataset pairs, for which a meaningful scoring is only possible with the XD-distance, are highlighted on the right. See also Table 1 for a list of the 20 top-ranked pathways in this plot

To score the association between a user-defined target gene/protein set and different reference datasets, the target set is first mapped onto a molecular network (here a connected human interactome graph extracted from the STRING 9.0 database (Snel *et al.*, 2000; Von Mering *et al.*, 2003), with edges weighted by the STRING combined confidence score normalized to range [0,1]). The network nodes corresponding to the target genes are then used as seed nodes for a random walk procedure to score their distances to all reference datasets. A random walk on a graph is a stochastic process modelling the iterative transition of an imaginary particle from a seed node in the graph to randomly chosen neighbour nodes over time. This enables the estimation of the proximity of a target node *t* to the seed node *s* by the steady-state probability with which the particle remains at node *t* (Fujiwara *et al.*, 2012). The motivation for using a random walk procedure as opposed to simpler distance measures like the shortest path distance is that by accounting both for the number and length of multiple pathways interconnecting two nodes, multi-facet relationships between them can be captured. Specifically, to enable the choice of an optimal trade-off between the exploitation of local and global network information, EnrichNet uses a random walk variant known as random walk with restart (Yin *et al.*, 2010), which allows the algorithm to restart the walk at the source nodes with probability *p* in every iteration (a pseudo-code version of the algorithm is shown in Fig. 1). The benefits of RWR for node relevance scoring have been discussed extensively in the literature (Tong *et al.*, 2008) and are already used in current approaches for disease-gene prioritization (Köhler *et al.*, 2008). To emphasize local neighbourhood information, EnrichNet runs the RWR algorithm using a high restart probability of *P* = 0.9. Importantly, in spite of its name, RWR is a deterministic procedure modelling a random walk via matrix computations (see Fig. 1). The random walk starts with equal probability from each of the genes in the target set and the generated relevance scores obtained for the nodes of the reference pathways are converted to distance scores by subtraction from 1, resulting in a distance score vector for each pathway. To relate these scores to a background model, the single distance score vectors are discretized into equal-sized bins and their deviations from the corresponding average distribution across all pathways is quantified by means of the Xd-distance, a distance measure that has previously been used in the evaluation of protein contact map predictions (Olmea *et al.*, 1999), defined as follows:
(1)
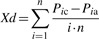

(2)
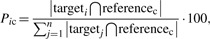

where *P_i_*_c_ is the percentage of distance scores for the target gene set and the current pathway *c* within bin *i* in relation to the total number of distance scores for pathway *c*, *P_i_*_a_ is the analogously defined percentage for the distance scores obtained across the background model of all pathways, *n* is the number of network distance bins (in our experiments, 10 distance bins provided sufficient sensitivity and are used as the default setting) and the current bin number *i* is used in the denominator to down-weight the score contribution of long distance and high-degree outliers, to prevent biases resulting from outstanding network properties of single genes/proteins. This weighting factor also accounts for the supposition that an over-representation of small distance scores is more likely to reflect strong associations than an over-representation of large distance scores. Classical statistical tests for comparing differences in the centre or shape of two distributions, e.g. the Mann–Whitney U-test or the Kolmogorov–Smirnov test, are not applicable in this context, because they lack a distance-dependent weighting. Similarly, random matched-size gene sets do not provide an adequate background model, since their members can only have similar connectivity properties as pathway-representing gene sets, if they are allowed to significantly overlap with real pathways in the network.

Apart from taking into account the information on the distances and the number of directly and indirectly connecting links between gene/protein sets in a molecular network, this scoring method also enables a straight-forward computation of tissue-specific association scores by only including the distances to nodes labelled for the tissue of interest in the reference datasets in the above calculation (in classical overlap-based enrichment analysis, a corresponding focussed tissue-specific analysis is often infeasible, because the intersection sets between the datasets become too small). Although the entire procedure is more computationally expensive than a classical over-representation analysis, this does not result in significant limitations for practical use, since an analysis takes only a few minutes for most pathway databases, and the web-interface optionally provides an e-mail notification for completed tasks. EnrichNet is also applicable to unweighted networks and a corresponding example network, as well as the possibility to upload user-defined networks, is provided on the web page.


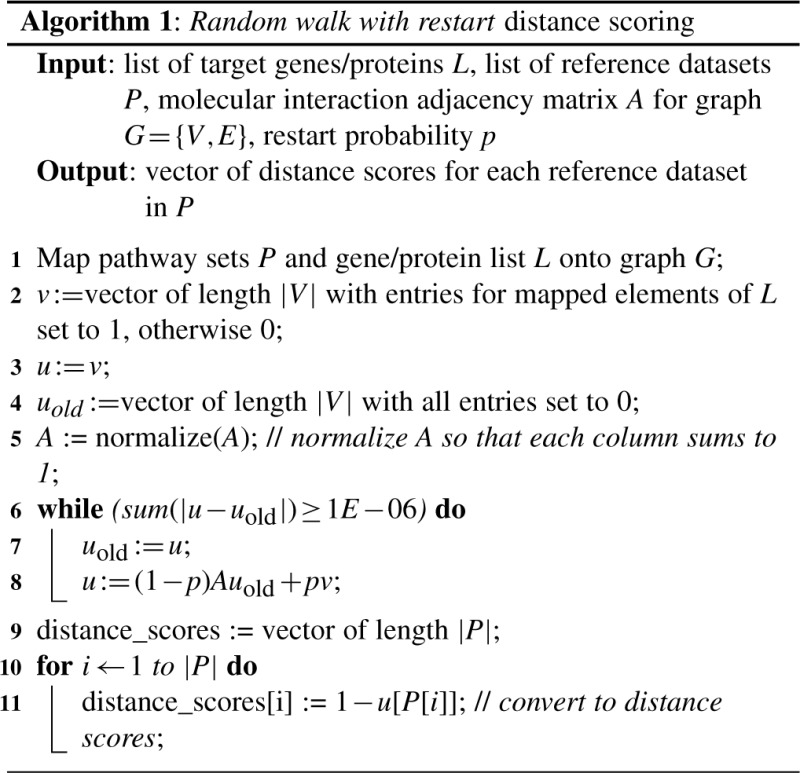


Finally, high observed correlations between the final Xd-distance association scores and classical over-representation scores for overlapping datasets, computed using Fisher's exact test and the method by Benjamini and Hochberg (1995) for multiple testing adjustment, are exploited to generate a regression plot (see Fig. 1) that enables the user to choose a significance threshold for the XD-scores which matches to a user-defined threshold for the adjusted *P*-value. The default threshold corresponds to an adjusted *P*-value of 0.05 with an additional increment given by the upper bound of the 95% confidence interval for linear regression fitting, added to account for the uncertainty in the fitted model parameters.

### 2.3 Evaluation method

To evaluate EnrichNet, we compare the approach with a classical ORA using Fisher's exact test (see Section 3.2) on all combinations between five labelled microarray gene expression datasets (p53 wild-type versus mutant cancer cell lines; Subramanian *et al.*, 2005), two lung cancer datasets with two outcome groups from a study conducted in Michigan (Beer *et al.*, 2002) and an independent study in Boston (Bhattacharjee *et al.*, 2001), a colon cancer dataset comparing tumour samples versus healthy controls (Alon *et al.*, 1999) and a dataset containing lower stage (Stages IA and IB) and higher stage (Stages IIB and III) cutaneous T-cell lymphoma samples (Shin *et al.*, 2007) and two frequently used reference gene set collections, *C1* and *C2* (Subramanian *et al.*, 2005). These datasets have been studied extensively in the literature and used to evaluate gene set enrichment analysis (GSEA) methods that take into account expression data for the estimation of pathway associations, but which (in contrast to the two methods compared here) are not applicable to gene and protein lists provided without additional expression measurements. Importantly, EnrichNet and ORA methods are designed specifically for the analysis of gene/protein lists that are not accompanied by any additional expression or activity measurements, and microarray data are used only for validation purposes. Specifically, the consensus of GSEA-derived pathway rankings is used as an external benchmark pathway ranking, exploiting the capability of GSEA methods to capture information from gene expression levels and combining two diverse GSEA approaches (see below). Similar evaluation techniques, using the combined evidence from multiple analysis methods and/or exploiting additional data sources to address the absence of a gold standard pathway ranking, have been used before, e.g. a recently introduced approach scored the extent to which gene sets ranked as significant by a method of interest are reproduced by other methods (Hung *et al.*, 2012). Here, we obtain the benchmark pathway rankings by first normalizing all the gene array datasets listed above using the ‘Variance Stabilizing Normalization’ approach (Huber *et al.*, 2002) and applying two recent GSEA methods, SAM-GS (Dinu *et al.*, 2007) and GAGE (Luo *et al.*, 2009), on all combinations of the microarray datasets with the reference gene set collections *C1* and *C2*. The resulting GSEA pathway rankings are then combined by computing the intersection sets between the 100 top-ranked pathways for each microarray/gene set collection pair (the specific number of pathways was chosen to obtain an equal-sized benchmark set across all datasets and reflects the observation that the estimated numbers of significant pathways at a *q*-value significance score cutoff of 0.05 across all methods and datasets cluster roughly around 100). To compare the EnrichNet and ORA pathway rankings against these benchmarks, first the top 100 most significantly differentially expressed genes (DEGs) according to the empirical Bayes moderated *t*-statistic (Smyth, 2004) are extracted for each microarray study and the significance scores adjusted for multiple testing according to Benjamini and Hochberg (1995) (again, the specific number of DEGs was chosen to obtain equal-sized target gene sets for all datasets and lies in between estimates for the number of significant DEGs at a *q*-value cutoff of 0.05). Next, the association scores between these DEGs and the gene set collections are computed using EnrichNet and ORA, providing two ranking lists for each microarray/gene set collection pair. The final evaluation scores are obtained by computing a running-sum statistic across the EnrichNet and ORA ranks for all gene sets from each reference collection, with positive score contributions for benchmark pathways and negative contributions for all other pathways, using the normalized Kolmogorov–Smirnov test as defined in Mootha *et al.* (2003).

## 3 RESULTS AND DISCUSSION

### 3.1 EnrichNet scores compared to over-representation analysis scores

A common biological application of enrichment analysis methods is the ranking of associations between a set of known disease-related genes and pre-defined gene/protein sets representing cellular pathways. To highlight the wide spectrum of potential biomedical applications, we assessed EnrichNet on two gene sets representing different tumour types (genes mutated in bladder and gastric cancer; Bamford *et al.*, 2004; Futreal *et al.*, 2004; Hamosh *et al.*, 2000) and one gene set representing a neurological disease (genes associated with Parkinson's disease; Yu *et al.*, 2010) and compared the results with a conventional over-representation analysis on all pathway databases.

In agreement with prior expectations, due to the dependency of both scores on the dataset overlap sizes, the network-based and the over-representation-based association scores (here using the Fisher's exact test) were highly correlated, with absolute Pearson correlations between 0.50 and 0.95 for the different datasets compared (see Fig. 1 for example; lower correlations can mainly be attributed to cases in which multiple pathways receive the same over-representation score but different Xd-scores, see Table 1 for the top 20 pathways in the correlation plot and paragraph below). Similar qualitative results were obtained with the Spearman correlation, with high absolute correlations overall, but lower absolute correlations in comparison to the Pearson measure.

**Fig. 2.**
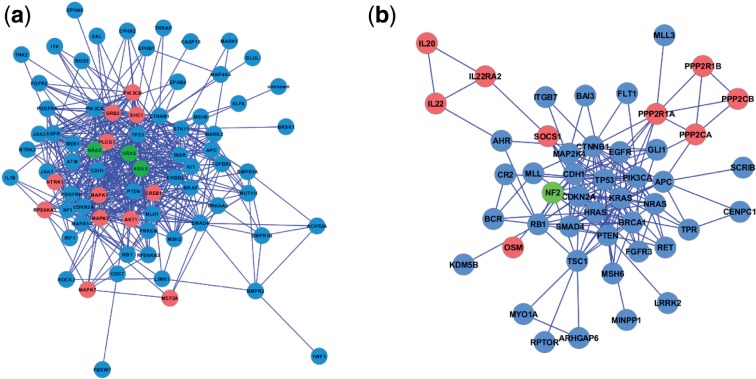
Protein–protein interaction sub-networks (largest connected components) for target and reference set pairs with small overlap, predicted to be functionally associated by EnrichNet: (a) gastric cancer mutated genes (blue) and genes/proteins from the BioCarta pathway ‘Role of Erk5 in Neuronal Survival’ (magenta, the shared genes are shown in green); (b) bladder cancer mutated genes (blue) and genes/proteins from Gene Ontology term ‘Tyrosine phosphorylation of Stat3’ (GO:0042503, magenta; the only shared gene *NF2* is shown in green). An over-representation analysis approach would have missed these associations, since only few of the cancer mutated genes are members of the corresponding processes

**Fig. 3.**
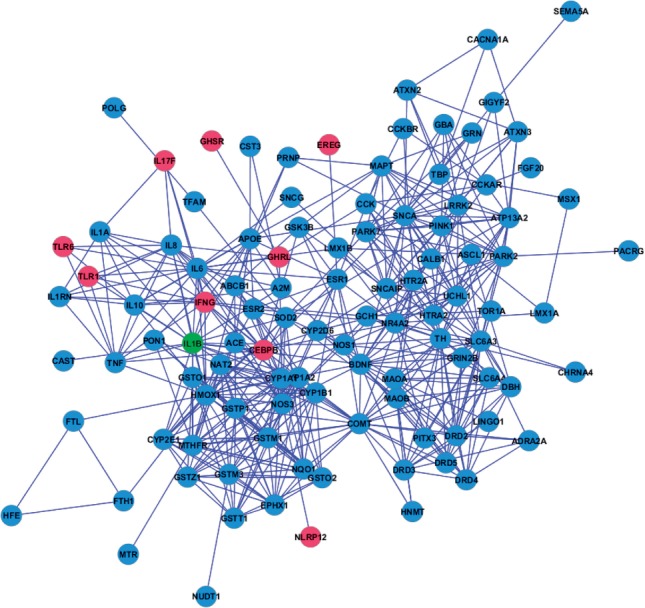
Protein–protein interaction sub-network (largest connected component) for the PD gene set (blue) and genes/proteins from GO term ‘Regulation of interleukin-6 biosynthetic process’ (magenta, GO:0045408; the only shared gene *IL1B* is shown in green)

More importantly, gene set pairs with equal over-representation scores (i.e. data points lying on the same vertical line in Fig. 1) can be differentiated using their Xd-distances. Both the datasets that share none of their genes/proteins with a pathway of interest (overlap size is zero) and cannot be scored with the over-representation approach (see right margin in Fig. 1) and those with large overlap-sizes and the same or similar overlap-based scores (see left part of Fig. 1) mostly receive different Xd-scores, enabling a more sensitive and comprehensive ranking of gene set pairs. For example, when scoring the BioCarta pathway associations of the gastric cancer mutated genes, seven different pathways receive the Fisher's exact test overlap score 0.01, whereas all their corresponding Xd-scores differ (see pathways highlighted in blue in Table 1). Although reference sets with empty or small intersection set with the target gene set will reach significant Xd-scores less frequently than datasets with large overlaps, cases with small or no overlap are particularly interesting for biological data interpretation, because they represent novel functional associations reflecting dense sub-networks of interactions rather than known associations of overlapping datasets (corresponding examples are shown in Section 3.3 and Figs 2 and 3). The Supplementary Materials provide additional tables with detailed molecular relations for further pathway/disease combinations across different databases.

### 3.2 Comparative validation on benchmark gene expression data

In order to evaluate EnrichNet quantitatively, pathway/gene set rankings were computed on all combinations of five benchmark microarray datasets and two gene set collections and compared against the results for a conventional ORA using Fisher's exact test, as described in Section 2. A set of high confidence benchmark pathways for each microarray/gene set collection pair was obtained by applying the recent gene set enrichment analysis methods SAM-GS and GAGE and computing the intersection set of between the 100 top-ranked pathways for each method. Table 2 shows the enrichment scores obtained when testing the over-representation of the benchmark pathways among the top-ranked entries in the EnrichNet and ORA rankings for all dataset combinations. Additionally, the table provides *P*-value significance score estimates for the enrichment scores, obtained in a non-parametric fashion using 1000 random permutations of the input rankings. In all cases, EnrichNet provides higher enrichment scores than the ORA approach and its *P*-value estimates are either lower or below the detection limit (0.001) for both methods. Considering the ‘No Free Lunch Theorem’ (Wolpert and Macready, 1995), these results do not prove a general superiority of the EnrichNet approach, but show that on common real-world datasets EnrichNet can reduce the gap in sensitivity between expression enrichment analysis methods like SAM-GS and GAGE and more generally applicable annotation enrichment analysis techniques, which are required in cases where only gene/protein lists and no expression data are available (see biological examples in the next section).

**Table 2.** Enrichment scores and *P*-value estimates for the comparative validation of EnrichNet and ORA using Fisher's exact test across all combinations of five microarray gene expression datasets and two gene set collections

Microarray dataset	Gene set collection	Fisher's exact test Enrichment score (*P*-value)	EnrichNet Enrichment score (*P*-value)
p53	C1	13.5 (*P* = 0.225)	36.9 (*P* < 0.001)
	C2	45.6 (*P* < 0.001)	65.2 (*P* < 0.001)
Lung (Boston)	C1	2.6 (*P* = 0.936)	40.0 (*P* < 0.001)
	C2	15.0 (*P* = 0.302)	43.7 (*P* < 0.001)
Lung (Michigan)	C1	21.2 (*P* = 0.028)	40.8 (*P* < 0.001)
	C2	9.1 (*P* = 0.634)	40.5 (*P* = 0.001)
Colon	C1	6.85 (*P* = 0.673)	70.1 (*P* < 0.001)
	C2	22.8 (*P* = 0.075)	94.9 (*P* < 0.001)
Lymphoma	C1	8.0 (*P* = 0.569)	65.2 (*P* < 0.001)
	C2	0.94 (*P* = 0.985)	69.8 (*P* < 0.001)

EnrichNet provides higher enrichment scores and lower or equivalent *P*-value estimates in all cases.

### 3.3 Identification of novel functional associations

In spite of the high correlations between the results for the network-based and the over-representation-based association measure (see Fig. 1), the Xd-score ranking identifies several new associations missed by the classical approach. Rather than studying the top-ranked pathways that receive both significant ORA scores and Xd-scores, the following examples therefore focus on dataset pairs with zero or insignificant overlap size (Fisher's exact test Q-value >0.05), which receive Xd-scores above the significance threshold obtained from the linear regression fit (see Section 2), since these results point to functional associations that reflect dense networks of interactions between the target and reference datasets, and are overlooked by approaches scoring only shared genes or proteins. Moreover, the used target gene sets all correspond to lists of genes that are mutated in different diseases without additionally available expression level data, i.e. they could not be analysed with microarray-specific gene set enrichment analysis techniques.

Two of these gene set associations detected by the EnrichNet methodology are visualized in Fig. 2. On the left (Fig. 2a), the largest connected component is displayed for the network structure obtained when comparing the gastric cancer mutated gene set against the pathway ‘Role of Erk5 (Extracellular signal-related kinase 5)’ in Neuronal Survival (h_erk5Pathway) from the BioCarta database, describing a signalling cascade which induces transcriptional events promoting neuronal survival. These datasets have an intersection of only three genes (*HRAS*, *NRAS* and *KRAS*—see green nodes in Fig. 2a) and would therefore not have been considered as significantly associated by an over-representation analysis using the Fisher's exact test (Q-value: 0.08). However, the obtained Xd-score (0.26, which matches with the regression fit based significance threshold), highlights functional associations reflecting the abundance of molecular interactions between the corresponding proteins for these gene sets and their shared network neighbourhood (instead of only their directly shared genes/proteins). This dense network of interactions corroborates previous findings linking extracellular signal-related kinases (ERKs) to gastric cancer via an induction of the putative tumour suppressor gene *DDMBT1* (deleted in malignant brain tumours 1) by a reduced *ERK*s activity (Kang *et al.*, 2005).

More interestingly, Xd-scores meeting the significance criterion were also obtained for dataset pairs with fewer shared genes or proteins. For example, Figure 2b shows the largest connected component in the network structure for two datasets, bladder cancer mutated genes (blue) and the genes for the Gene Ontology (GO) term ‘tyrosine phosphorylation of Stat3 (GO:0042503)’, which share only a single gene (*NF2*) and for which no association can be inferred from an over-representation analysis. The high Xd-score for this gene set pair (0.80, the significance threshold is 0.45) points to a functional association via multiple connecting molecular interactions, which is confirmed by the visualization. This result is in agreement with the previously reported observation that the down-regulation of *STAT3* phosphorylation by means of silencing the Rho GTPase *CDC42* is linked to the suppression of tumour growth in bladder cancer (Wu *et al.*, 2008). Rho GTPases like *CDC42* are known to frequently participate in carcinogenic processes (del Pulgar *et al.*, 2005) and their involvement in bladder cancer is also reflected by a high Xd-score of 0.71 for the GO biological process ‘regulation of Rho GTPase activity’ (GO:0032319), which also shares only one gene with the bladder cancer mutated genes (*TSC1*).

For the third gene set, containing genes implicated in Parkinson's disease (PD) (Yu *et al.*, 2010), EnrichNet found a strong association with the ‘regulation of interleukin-6 biosynthetic process’ from the Gene Ontology database (GO:0045408, see Fig. 3. The pathway is ranked with a significant XD-score (0.77, significance threshold: 0.73) and shares only one gene (*IL1B*) with the PD dataset, preventing the identification of a functional association by means of a conventional over-representation analysis (Fisher's Q-value: 0.55). The visualization of the corresponding sub-network (see Fig. 3) reveals a dense cluster of interactions that interlink the PD gene set with the interleukin-6 pathway. This gene set association corroborates previously identified links between PD and inflammation (Knott *et al.*, 2000) and reports of elevated levels of interleukin-6 in the cerebrospinal fluid of PD patients (Blum-Degena *et al.*, 1995).

In summary, these example applications of the network-based scoring methodology illustrate the utility of the approach for identifying novel functional associations between gene/protein sets, which reflect known direct and indirect molecular interactions between their members rather than only the size of their overlap. Further novel associations identified for these cancer datasets and PD are presented in the Supplementary Materials.

### 3.4 Evaluation of the tissue specificity of gene set associations

The Xd-distance is capable of computing tissue-specific association scores (see Section 2). Although tissue-specific analyses can also be realized with other enrichment analysis techniques, in particular methods that enable the consideration of non-overlapping genes/proteins through additional expression level measurements or an extension of the target and reference gene sets, a corresponding analysis is in practice often infeasible for conventional ORA methods, which are applicable to fixed gene/protein lists without complementary expression measurements. This practical limitation results from the typically small size of the intersection set between the target and reference dataset, because the subset of genes with available tissue-specific annotations within the intersection set of genes/proteins is often too small for at least some of the analysed tissues to obtain reliable over-representation statistics. EnrichNet alleviates this limitation of ORA approaches by additionally taking tissue specificity annotations into account for all non-overlapping gene/protein pairs, which are connected through paths of interactions in a molecular network. We illustrate the informative value of EnrichNet's tissue-specific scores using a comparative analysis of brain and non-brain tissues (see the details on the tissue grouping for 60 human tissues in the Supplementary Materials). Specifically, we apply EnrichNet on a set of genes with known implications in PD (Yu *et al.*, 2010) and measure the tissue-specific associations with the high-scoring KEGG ‘Neurodegenerative Diseases’ (hsa01510) pathway. As expected, high Xd-scores were over-represented in the group of brain tissues, whereas the centre of the Xd-score distribution was significantly lower in the non-brain tissues (P = 0.004, Mann–Whitney test). This example highlights the utility of the scoring scheme in providing information to identify tissue-specific associations between genes/proteins in molecular interaction networks and to rule out associations with low Xd-scores in a tissue of interest.

*Funding:* The Biotechnology and Biological Sciences Research Council (BB/F01855X/1), and the EU FP7 project Microme (grant number 222886) the Spanish Ministry for Education and Science (BIO2007-66855). N.K. is supported with an Engineering and Physical Sciences Research Council Leadership Fellowship (EP/J004111/1) and a Morris Belkin visiting professorship at the Weizmann Institute of Science.

*Conflict of Interest:* none declared.
